# Progressive Soft Tissue Release and Latarjet Procedure in the Treatment of Neglected Anterior Shoulder Dislocation After Failed ORIF in a Young Active Patient: A Case Report

**DOI:** 10.1155/cro/2605783

**Published:** 2026-09-30

**Authors:** Juan David Lacouture, Vanessa Otoya-Giorgi, Henry Alejandro Pulido González, Jorge Rojas Liévano, Juan Carlos Gonzalez-Gomez, Guido Fierro-Porto

**Affiliations:** ^1^ Department of Orthopaedics and Traumatology Shoulder and Elbow Section Hospital Serena del Mar Cartagena Colombia; ^2^ Department of Orthopaedics and Traumatology Shoulder and Elbow Section Fundación Santa Fe de Bogotá Bogotá Colombia fsfb.org.co; ^3^ School of Medicine Universidad de Los Andes Bogotá Colombia uniandes.edu.co

**Keywords:** anterior shoulder instability, case report, Latarjet procedure, neglected shoulder dislocation, open reduction

## Abstract

**Background:**

Neglected anterior shoulder dislocation (NASD) is rare, representing less than 2% of all shoulder dislocations, and poses a complex surgical challenge driven by chronic soft tissue contracture, bone loss, and risk of humeral head collapse. Most evidence derives from case series in elderly patients, and reports detailing progressive soft tissue release strategies in young active individuals remain scarce.

**Case Presentation:**

A 25‐year‐old male kitesurfer presented with an 8‐month history of NASD following unsuccessful open reduction and internal fixation for a proximal humeral fracture‐dislocation. The patient had marked limitation in range of motion and visible deformity, without axillary nerve dysfunction, with imaging findings of 30% glenoid bone loss. Surgical management consisted of progressive soft tissue release and controlled open reduction, including coracoid osteotomy, upper two‐thirds subscapularis tenotomy, supraspinatus release, and posterior capsulotomy, followed by a conventional Latarjet procedure. At 24‐month follow‐up, the patient demonstrated significant improvement in range of motion, patient‐reported outcomes (Constant 92, ASES 95), complete pain relief, maintained joint stability, and partial return to recreational kitesurfing, without complications.

**Conclusions:**

A structured approach based on progressive soft tissue release combined with stepwise open reduction is essential to safely restore joint congruency in NASD. In selected young patients with significant glenoid bone loss, the Latarjet procedure provides stability and supports favorable functional outcomes.

## 1. Introduction

Neglected anterior shoulder dislocations (NASDs) are chronic dislocations that remain unreduced for more than 3 weeks [1–3]. They represent a rare clinical entity, comprising less than 2% of all shoulder dislocations with prevalence around 0.10%–0.18% [2, 4]. NASDs are more frequently encountered in elderly patients, those with seizure disorders or alcohol use, and in low‐resource settings [2, 4, 5]. Their complexity is driven by a pathoanatomic triad: (1) anterior neoglenoid formation with glenoid and humeral bone loss, (2) significant soft tissue contracture involving the capsule and rotator cuff, and (3) increased risk of humeral head collapse [1, 3, 6].

Surgical management ranges from open reduction alone to bone block techniques such as the Latarjet procedure [1, 3, 6, 7]. Systematic reviews have demonstrated high resubluxation and early arthritis rates after open reduction, with substantial variation in outcomes, and nearly all available data are Level IV–V evidence [3]. Conservative management generally yields poor functional results due to soft tissue interposition and bony block [3, 5]. A 30% major complication rate has been reported when associated factors such as greater tuberosity fractures or advanced rotator cuff fatty infiltration are present [1].

Despite growing literature on NASD, most reports focus on elderly patients or those with low functional demands. We present a case detailing progressive soft tissue release and Latarjet procedure in a young athlete, with emphasis on reproducible technique and midterm clinical outcomes.

## 2. Case Report

### 2.1. Patient Information and Timeline

A 25‐year‐old right‐hand‐dominant male patient, who practices kitesurfing recreationally twice per week, sustained a traumatic injury to the right shoulder during a sports activity. The mechanism involved abduction and external rotation, resulting in immediate deformity. Initial evaluation at an outside institution revealed a proximal humerus fracture‐dislocation with displacement of the greater tuberosity. The patient reported that following the initial surgery, he noted recurrent instability of the right shoulder but remained functionally able to perform daily activities without limitation. As a recreational kitesurfer, he chose initially to modify his activities, avoiding the sport and attempting conservative self‐management with the expectation of spontaneous resolution. However, after 8 months of progressive pain and persistent restriction in range of motion despite activity modification, he recognized that the condition was unlikely to improve without professional intervention and therefore presented to our institution for evaluation and management (Table 1). The patient is a nonsmoker; family and psychosocial histories were unremarkable and not considered relevant to the clinical presentation or management. Written informed consent was obtained from the patient for publication of this case report and accompanying images; afterwards, institutional ethics committee approval was requested and granted with the following approval number CCEI‐19692‐2026.

**Table 1:** Clinical timeline.

Time point	Clinical event
Day 0	Initial trauma: Proximal humerus fracture‐dislocation during kitesurfing
Week 1	ORIF with cannulated screws (transdeltoid approach) at outside institution
Weeks 2–4	Suspected recurrent dislocation (not evaluated); progressive ROM limitation
Month 8	Presentation to our institution; NASD diagnosis confirmed
Months 8 + 2 weeks	Definitive surgery: Progressive soft tissue release and Latarjet procedure
Month 32 (24 months after surgery)	Final follow‐up: Constant 92, ASES 95, stable joint, no complications, partial return to recreational kitesurfing

### 2.2. Clinical Findings

Eight months after the initial intervention, the patient presented at our shoulder and elbow unit at a Level I trauma center with persistent functional limitation. Pain was 6/10 on the visual analog scale (VAS) during physical activity, with a subjective shoulder value (SSV) of 40% and complete inability to participate in sports. Physical examination revealed loss of the lateral shoulder contour with the humeral head palpable anteriorly (Figure 1A). Active range of motion of the affected right shoulder was severely limited compared with the normal contralateral left shoulder: Forward elevation was 60° on the right versus 160° on the left, external rotation at 0° of abduction was 10° on the right versus 60° on the left, and internal rotation reached the iliac crest on the right versus L3 on the left. Deltoid strength was preserved (5/5 on the Medical Research Council [MRC] scale) with no clinical evidence of axillary nerve dysfunction.

**Figure 1:**
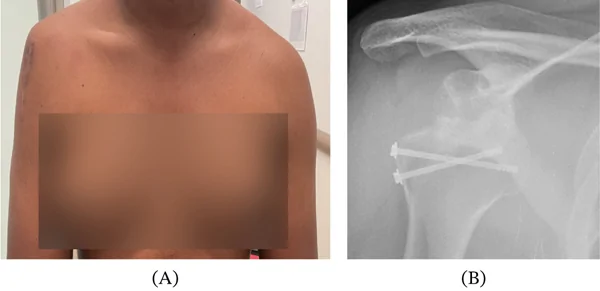
(A) Clinical photograph demonstrating loss of lateral shoulder contour with anterior prominence. (B) Preoperative anteroposterior radiograph showing neglected anterior shoulder dislocation with cannulated screws from prior greater tuberosity fixation.

### 2.3. Radiograph Assessment

The patient had a chronic unaddressed NASD (Figure 1B) associated with 30% glenoid bone loss, measured using the best fit circle method (Figure 2D). A neoglenoid had formed and was articulating with the humeral head (Figure 2C). The Hill–Sachs lesion was assessed on axial imaging at the level of maximal defect, demonstrating a width of 2.3 cm and a depth of 1.0 cm, considering an off‐track lesion, absence of osteophytes, or bone cysts (Figure 2A,B).

**Figure 2:**
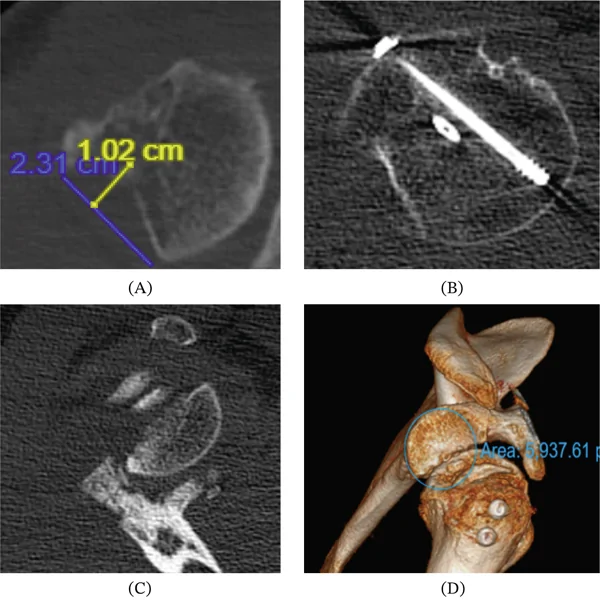
Computed tomography. (A) Axial view with Hill–Sachs lesion of 2.3 cm width × 1 cm depth. (B) Axial view with identification of screws in the correct position. (C) Axial view demonstrating anterior position of the humeral head with neoglenoid formation. (D) Three‐dimensional reconstruction showing 30% glenoid bone loss (best fit circle method).

### 2.4. Surgical Technique

A progressive open reduction combined with a modified Latarjet procedure for the management of a chronic locked anterior glenohumeral dislocation was performed. The patient was placed in the lazy beach chair position under general anesthesia combined with an interscalene brachial plexus block for intraoperative and postoperative analgesia. A standard deltopectoral approach was used. Given the chronicity of the dislocation and the fixed subcoracoid position of the humeral head, the surgical strategy was deliberately sequenced to achieve progressive soft tissue release before attempting reduction. While the overall construct mirrors a conventional Latarjet procedure, the critical difference lies in the extensive circumferential soft tissue release and humeral head reduction that must be accomplished between the coracoid osteotomy and the final bony fixation steps—stages that are not required in a standard Latarjet. The technique was performed in the following steps:

1. Coracoid osteotomy
2. Progressive soft tissue release
  o. Subcoracoid, subdeltoid, and subacromial bursectomy
  o. Upper subscapularis tenotomy
  o. Glenohumeral fibrosis release
  o. Posterior capsulotomy
3. Humeral head reduction
4. Latarjet procedure
  o. Glenoid preparation
  o. Coracoid fixation
  o. Subscapularis reinsertion

### 2.5. Coracoid Osteotomy

As in a standard Latarjet procedure, the coracoid osteotomy was performed as the first step. The coracoacromial ligament and pectoralis minor tendon were released from the coracoid medially and laterally, and the coracoid was osteotomized at its base. In this context, however, the osteotomy served an additional purpose beyond graft harvesting: It created the necessary working space and surgical exposure required to perform the subsequent extensive soft tissue release maneuvers, which would not have been safely achievable with the coracoid in situ given the fixed subcoracoid malposition of the humeral head.

### 2.6. Progressive Soft Tissue Release

This step represents the key departure from a routine Latarjet procedure and was essential to safely mobilize the chronically dislocated humeral head prior to reduction. In a standard Latarjet, soft tissue handling is limited to a subscapularis split or tenotomy; in this case, extensive circumferential release was required and performed in a deliberate, sequential fashion to avoid neurovascular injury and minimize the risk of iatrogenic humeral head damage. First, a subcoracoid bursectomy was performed, followed by complete subacromial and subdeltoid bursal release, providing circumferential visualization of the rotator cuff. The subscapularis, supraspinatus, and posterosuperior cuff at the level of the infraspinatus were carefully identified and fully mobilized. Subsequently, the upper two‐thirds of the subscapularis tendon were tenotomized, with the tendon tagged for later reinsertion. This allowed direct exposure of the glenohumeral joint. Dense fibrosis involving the posterior glenoid and supraspinatus was carefully excised, and a complete posterior capsulotomy was performed to eliminate any residual soft tissue tether preventing reduction; posterior release was performed under direct visualization, close to the glenoid rim and intra‐articular plane, after circumferential mobilization, while avoiding blind posterior dissection and inferior extension near the axillary nerve. At the completion of this step, circumferential release had been achieved, and the humeral head was free to be reduced.

### 2.7. Humeral Head Reduction

With complete soft tissue release accomplished, gentle traction‐countertraction, and controlled extra‐articular leverage at the humeral neck/metaphyseal region, instrumentation was deliberately avoided on the articular surface to prevent iatrogenic chondral damage and reduce the risk of secondary humeral head collapse—a recognized complication in the management of chronic locked dislocations.

### 2.8. Latarjet Procedure

Following successful reduction, the Latarjet procedure was completed in a conventional manner. The anterior glenoid rim and the coracoid contact surface were decorticated and abraded to optimize bony contact and promote union. The coracoid graft was positioned at the anterior glenoid face, flush with the articular surface, and secured with two parallel cannulated screws oriented perpendicular to the glenoid face. Screw positioning and graft alignment were confirmed fluoroscopically. Full passive range of motion was verified intraoperatively to ensure no impingement. Finally, the subscapularis tendon was reinserted to its native footprint, restoring the anterior soft tissue restraint.

### 2.9. Follow‐Up and Outcomes

Postoperatively, the patient was immobilized in a sling. Rehabilitation included Codman′s pendulum exercises during Week 1, passive mobility from Week 2 with external rotation restricted until Week 6, and then progressive strengthening. At 1 month, complete passive mobility was achieved (forward flexion 160°, external rotation 0°, and abduction 45°). After Week 6, rehabilitation progressed to active‐assisted and active range‐of‐motion exercises, followed by progressive strengthening and sport‐specific rehabilitation. Radiographs confirmed proper glenohumeral reduction and coracoid positioning (Figure 3A,B).

**Figure 3:**
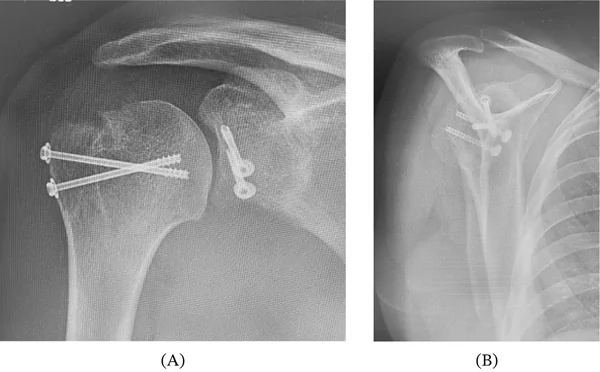
Follow‐up. (A, B) One‐month postoperative anteroposterior radiograph demonstrating concentric glenohumeral reduction with proper positioning of the coracoid autograft and fixation screws.

At 24‐month follow‐up, the patient demonstrated complete pain relief (VAS 0/10), SSV of 90%, and active mobility of forward flexion 140°, external rotation 40°, and internal rotation to T7 (Figure 4A,B). Functional scores were excellent (Constant 92 and ASES 95). Painless functional range of motion, absence of apprehension or instability, radiographic graft consolidation, and satisfactory strength compared with the contralateral side were considered before authorization for return to sports. After partial return to sports activities, he resumed recreational athletic participation; however, he did not return to competitive sports or to his preinjury level of performance. CT demonstrated complete coracoid graft consolidation, concentric reduction, no osteoarthritis changes, and proper screw positioning (Figure 4C,D). No adverse events occurred.

**Figure 4:**
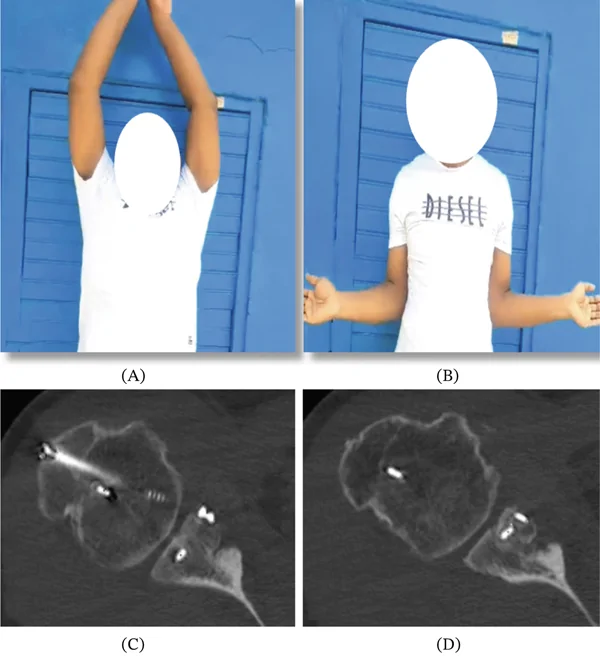
Twenty‐four‐month follow‐up clinical images and computed tomography. (A) Forward flexion 140°. (B) External rotation 40°. (C, D) Axial view showing concentric reduction, complete coracoid graft consolidation, and absence of secondary arthritis.

### 2.10. Patient Perspective

From the patient′s perspective, the injury occurred after a fall from standing height and resulted in severe functional limitation of his shoulder. He reported a rapid and progressive recovery after surgery and rehabilitation. At final follow‐up, he was able to perform all daily activities normally and had returned to recreational sports, including kitesurfing. The patient expressed satisfaction with the functional recovery achieved.

## 3. Discussion

NASD treatment presents several challenges: variety of management options, technical complexity driven by the pathoanatomic triad, and steep learning curve given its rarity (< 2% of dislocations) [2–4].

Conservative management yields poor functional results [3, 5]. Babalola et al. and Rowe and Zarins reported low outcomes with nonsurgical treatment, though for patients with low functional demand, conservative management may be viable [2, 8, 9]. Among surgical options, Abdelhady reported constant scores of 74 ± 5.2 with remplissage and Putti–Platt procedures [10]. Benabdallah and Khamlichi′s series of 53 shoulders—one of the largest reported—demonstrated mostly good results with head‐preserving open reduction, but with nontrivial rates of instability and infection [5].

Treatment with Latarjet has been controversial. Domos et al. reported it as relatively contraindicated due to complication risk [11]. However, multiple series support Latarjet for chronic dislocation with significant glenoid bone loss, showing good functional outcomes and low recurrence [4, 7, 12–16]. Sahu and Gupta proposed a classification: Type 1 patients (no severe associated injuries) showed favorable outcomes, while Type 2 patients (greater tuberosity fractures or advanced fatty infiltration) had a 30% rate of major complications [1]. Based on these considerations, in Type 1 NASD with significant glenoid bone loss (> 25%), the Latarjet procedure may be indicated in young patients; we considered the Latarjet procedure in our patient because of available local autograft, dynamic sling effect, and absence of severe associated injuries, but we also acknowledged that alternative bone‐block options may be considered as an option.

Regarding technique, we emphasize progressive soft tissue release as critical for safe reduction. Chronic retraction and fibrosis require stepwise release: subcoracoid, subacromial, and subdeltoid spaces, followed by superior two‐thirds subscapularis tenotomy. This systematic approach minimizes tension on the humeral head and may reduce postreduction collapse risk.

This report contributes to limited NASD literature. First, the patient represents an atypical demographic—a young, highly active athlete—whereas most series focus on elderly patients [2, 4, 5]. Second, successful outcome following failed primary ORIF demonstrates technique applicability in revision scenarios. Third, functional outcomes (Constant 92 and ASES 95) exceed those typically reported (Constant 69–74) [1, 10]. Fourth, absence of complications contrasts with the 30% rate reported in Type 2 cases [1]. Finally, return to high‐demand sport is rarely documented in NASD literature.

As a single case report, generalizability is limited, and nearly all NASD literature consists of Level IV–V evidence without standardized protocols [3]. The prior surgical intervention may have influenced outcomes. Long‐term outcomes beyond 24 months reported osteoarthritis progression at high rates in systematic reviews [3]. From the patient′s perspective, the intervention resulted in a meaningful recovery. At 24 months of follow‐up, the patient reported significant pain relief, restoration of daily activities, and satisfaction with the functional outcome, noting that the inability to use his dominant arm had severely impacted his quality of life prior to surgery.

## 4. Conclusion

NASD is rare and technically demanding, characterized by bone loss, soft tissue contracture, and humeral head collapse risk. Progressive soft tissue release with stepwise open reduction safely restores joint congruency. In selected young patients with significant glenoid bone loss and without severe associated injuries, the Latarjet procedure provides stability and favorable functional outcomes, including potential return to recreational sport.

## Funding

No funding was received for this manuscript.

## Consent

Written informed consent was obtained from the patient for publication of this case report and accompanying images. Patient consent was obtained; afterwards, institutional ethics committee approval was requested and granted with the following approval number CCEI‐19692‐2026.

## Conflicts of Interest

The authors declare no conflicts of interest.

## Data Availability

Data are available on request due to privacy/ethical restrictions.
